# The Efficacy of Modified Laparoscopic Burch Procedure Using a Single Stitch on Each Side of the Urethra for the Treatment of Stress Urinary Incontinence

**DOI:** 10.3390/medicina61030436

**Published:** 2025-02-28

**Authors:** Marilena Pirtea, Laurențiu Pirtea, Simona Brasoveanu, Ligia Balulescu, Flavius Olaru, Dragos Erdelean, Cristina Secosan, Dan Navolan

**Affiliations:** Department of Obstetrics and Gynecology, Victor Babes University of Medicine and Pharmacy, 300041 Timisoara, Romania; marilena.pirtea@umft.ro (M.P.); simona.brasoveanu@umft.ro (S.B.); ligia.balulescu@umft.ro (L.B.); olaru.flavius@umft.ro (F.O.); erdelean.dragos@umft.ro (D.E.); secosan.cristina@umft.ro (C.S.); navolan@umft.ro (D.N.)

**Keywords:** modified laparoscopic Burch procedure, Urinary Distress Inventory-6, Patient Global Impression of Improvement, Incontinence Severity Index

## Abstract

*Background and Objectives*: This study aimed to evaluate the efficacy and safety of modified laparoscopic Burch intervention over a 24-month follow-up period. *Materials and Methods*: We performed a retrospective cohort evaluation including all eligible patients, 83 patients, who underwent modified laparoscopic Burch colposuspension for stress urinary incontinence (SUI). Primary outcomes included the presence or absence of SUI on follow-up and the success of index surgery based on responses to validated questionnaires of patient-reported outcomes. *Results*: Patient-reported outcomes indicated a progressive improvement in perceived well-being over time. At the 6-month follow-up, 50.6% of participants reported their condition as “greatly improved”, increasing cumulatively to 66.7% by 24 months. The severity of urinary incontinence symptoms was markedly reduced following the intervention. The incidence of severe incontinence was notably low, with only 4.8% of patients affected at 6 months, remaining consistent at 5.1% at 24 months. This finding aligns with a high procedural success rate, as the vast majority of patients (≥94.9%) reported no severe symptoms across all follow-up intervals. Dryness, defined as the absence of urinary leakage, demonstrated an upward trend over time. At 6 months, 45.8% of patients reported complete dryness, with this figure rising to 55.1% at 12 months and 62.8% at 24 months. The Urogenital Distress Inventory-6 (UDI-6) served as a critical metric for evaluating the subjective burden of urinary symptoms. Across all follow-up intervals, over 97% of patients achieved scores below the clinically significant threshold (<33), indicating substantial symptom relief and enhanced quality of life. *Conclusions*: The modified laparoscopic Burch colposuspension demonstrated consistent efficacy, with significant improvements in urinary continence, symptom severity, and quality of life over the 24-month follow-up period.

## 1. Introduction

The prevalence of stress urinary incontinence (SUI) in women increases with advancing age, ranging from 14.8% to 31.8% among women over the age of 50, with a notable rise in prevalence during early adulthood [1].

The Burch procedure, first reported in 1961, remained the most widely used technique for SUI until the introduction of the suburethral sling by Petros P. and Ulmsten U. in 1995 [2].

Midurethral slings (MUSs) have been successfully used for the last 20 years in the treatment of SUI and have become the gold-standard surgical procedure. The transobturator tape procedure (TOT), also known as the “outside-inside” technique, was first described by Delorme in 2001 and has demonstrated a high rate of success with low perioperative complications. Consequently, the Burch colposuspension procedure has been largely abandoned in practice in many centers worldwide [3,4].

In 2019, the Food and Drug Administration (FDA) banned the utilization of transvaginal meshes for pelvic organ prolapse (POP), a restriction that affected the use of synthetic meshes for incontinence [5].

SUI is often found to coexist with POP. A study by Bai et al. reported that 63.3% of patients with SUI also had POP, most commonly affecting the anterior wall. Additionally, 62.7% of patients with POP had concurrent SUI [6].

Concomitant prolapse surgery, particularly anterior colporrhaphy, has been identified as an independent risk factor for postoperative voiding dysfunction following surgical treatment of SUI. The limited mobility of the vaginal wall after anterior prolapse surgery, as well as potential nerve injury, may contribute to failed or delayed postoperative voiding [7].

In July 2018, the UK government implemented a temporary suspension of the use of vaginally inserted surgical mesh for SUI due to safety concerns. Due to growing concerns over a complete ban of transvaginal tapes for incontinence in the future, gynecologists have rediscovered the Burch colposuspension for treatment of patients with SUI [8]. The procedure is experiencing a renaissance, and the shift away from mesh implants necessitates that surgical training programs reintroduce and emphasize proficiency in procedures like the Burch colposuspension to ensure that new surgeons are adequately prepared to offer alternative treatments for SUI [2].

Modified versions of the original technique described by Burch have been proposed, involving minimally invasive approaches aimed at ensuring faster recovery and a more standardized procedure. Variants using one or two paraurethral sutures have been suggested. The effectiveness of these techniques, as well as the associated complication rates, has been evaluated [2].

We chose to use a single stitch because we wanted to reinforce only the middle third of the urethra. Given the short length of the urethra, placing two stitches would extend the reinforced area toward the bladder neck and could alter the angle between the urethra and bladder neck. Our objective was to mimic the effect of the TOT procedure, which reinforces the middle third of the urethra.

Voiding dysfunction, a known complication of anti-incontinence surgery, may arise due to overcorrection of the urethra caused by excessive elevation of the bladder neck. Studies have reported that up to 20% of patients experience postoperative voiding difficulties or urinary retention following Burch colposuspension [9]. This condition encompasses a range of symptoms, from obstructive voiding and complete urinary retention requiring intermittent catheterization to irritative storage symptoms such as de novo urgency and detrusor overactivity. While mild cases of voiding dysfunction often resolve with conservative management, symptoms persisting beyond one month rarely resolve spontaneously [10].

The modified Burch technique, in contrast to the traditional Burch technique, aligns with the principles of anatomical restoration outlined in Petros’ Integral Theory [11]. This alignment is consistent with the low incidence of de novo urgency and voiding difficulties observed in patients who underwent the modified laparoscopic Burch technique [12].

Objectives: The study aimed to evaluate the efficacy and safety of modified laparoscopic Burch intervention over a 24-month follow-up period.

## 2. Materials and Methods

### 2.1. Study Design

We performed a retrospective cohort evaluation including all eligible patients, 83 patients, who underwent modified laparoscopic Burch colposuspension for SUI in the Department of Obstetrics and Gynecology of Timișoara University City Hospital, between January 2018 and December 2022.

This study was conducted after receiving approval from the Human Ethical Committee of the Victor Babes University of Medicine and Pharmacy, Timisoara, Romania (Approval Number: 58/12 December 2017), in strict adherence to ethical standards. All interventions carried out in this study involving human participants conformed to the principles set forth in the Declaration of Helsinki (revised in 2013). Written informed consent was obtained from all patients prior to participation.

### 2.2. Patient Selection

**Figure 1:**
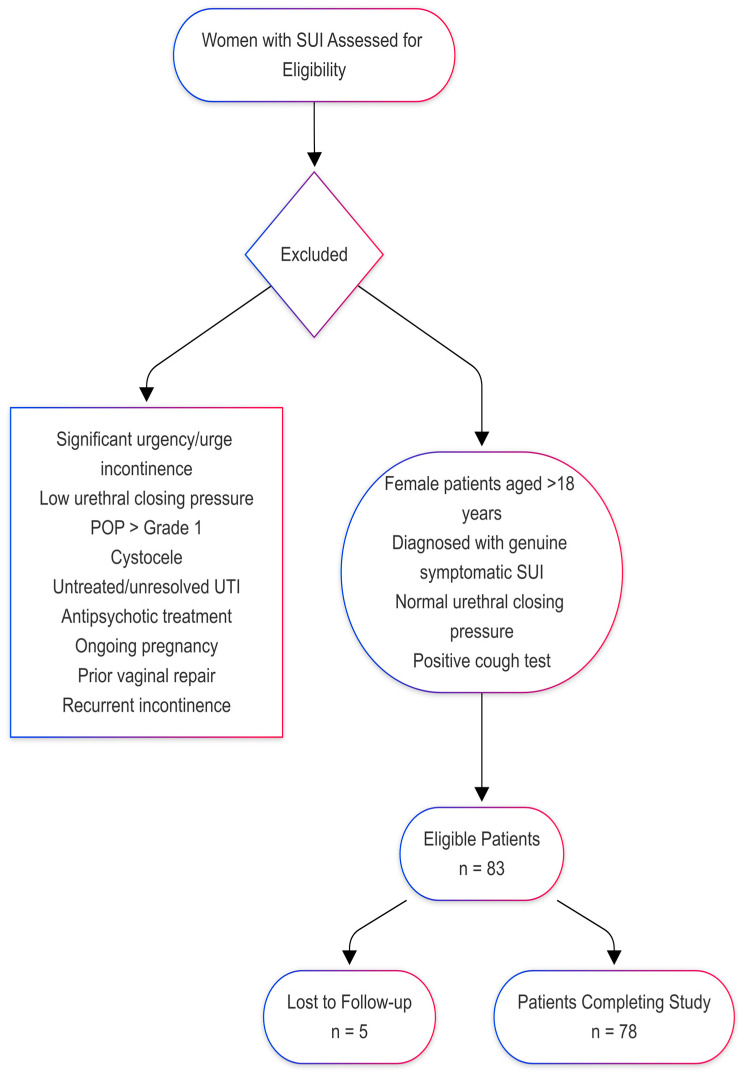
PRISMA flowchart outlining the patient selection process for the study on modified laparoscopic Burch colposuspension for stress urinary incontinence (SUI). A total of 83 eligible patients were included, with exclusion criteria based on clinical and comorbidity factors. Five patients were lost to follow-up, leaving 78 patients completing the study.

Women with SUI may also experience voiding dysfunctions, such as overactive bladder, dysfunctional voiding, detrusor underactivity, or increased post-void residual volume. These conditions can influence treatment outcomes. As shown in Figure 1, the inclusion criteria were female patients over 18 years of age with genuine symptomatic SUI, normal urethral closing pressure, and a positive cough test.

The exclusion criteria comprised female patients with urinary incontinence accompanied by significant urgency or urge urinary incontinence as identified through a bladder diary, as well as SUI resulting from low urethral closing pressure. Additionally, patients with comorbidities likely to impact the results, such as POP greater than grade 1, cystocele, untreated or unresolved urinary tract infection, or those undergoing antipsychotic treatment (due to the risk of urinary retention), were excluded. Ongoing pregnancy, prior vaginal repair, or recurrent incontinence were also excluded. We chose to exclude these patients because we did not want to incorporate additional POP reconstructive techniques. In our setting, for patients with SUI diagnosed through a cough test, urodynamic testing is not performed.

### 2.3. Outcomes of the Study

Primary outcomes included the presence or absence of SUI at follow-up and the success of the index surgery, based on the responses of the patients to validated questionnaires. Additional variables, such as age, parity, and baseline symptom severity, were included to assess their potential impact on outcomes.

### 2.4. Surgical Technique

The procedures were performed by the same surgical team, and a single dose of prophylactic antibiotics was administered preoperatively. The Foley catheter was removed 24 h after the modified laparoscopic Burch procedure.

The step-by-step technique for the modified laparoscopic Burch procedure is detailed below.

Standard laparoscopic instruments used include bipolar forceps, scissors, atraumatic grasping forceps, and two needle holders. For suturing, we used a 2/0 monofilament non-absorbable thread with a 3/8, 26 mm round needle for the Cooper ligament and a 2/0 absorbable (Vicryl) suture for the parietal peritoneum. Pneumoperitoneum was established using direct entry. To clearly expose the bladder dome’s upper limit and prevent injury, the bladder was filled with 150–200 mL of saline solution before making the peritoneal incision. A transperitoneal approach to the Retzius space was created by making a 5–6 cm incision in the parietal peritoneum, located between the obliterated umbilico-vesical arteries above the bladder dome. Once the peritoneum was incised, the bladder was drained, and the avascular space of Retzius was opened through blunt dissection.

The anatomical landmarks include the pubic symphysis and Cooper’s ligaments, with the Foley catheter used as a reference to locate the bladder neck.

**Figure 2:**
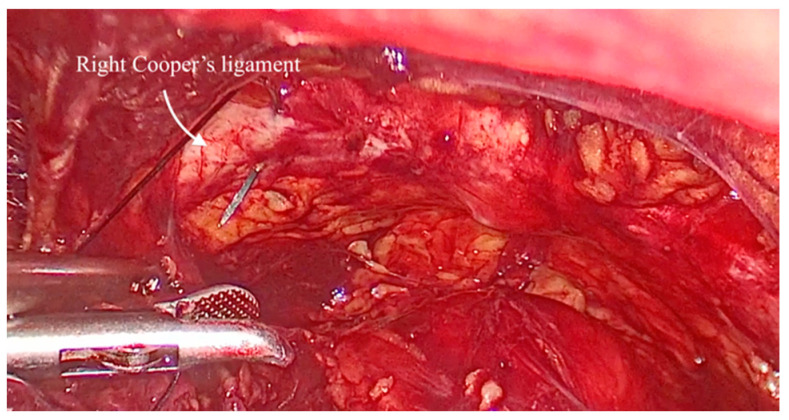
The aspect view of the space of Retzius: iliopectineal line (also known as Cooper’s ligament).

**Figure 3:**
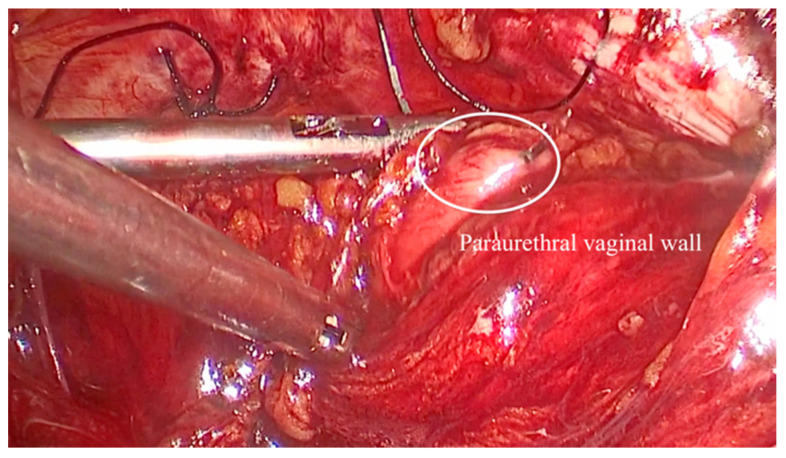
The aspect of paraurethral vaginal wall in the middle-third part of the urethra.

**Figure 4:**
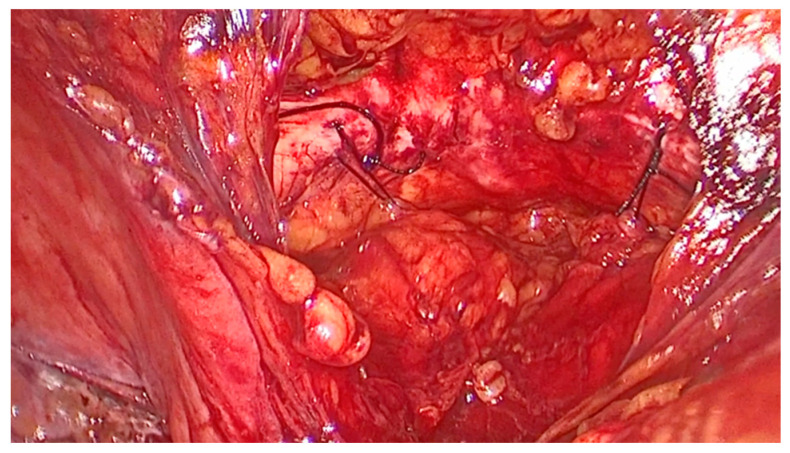
The final aspect of modified laparoscopic Burch colposuspension technique with one thread on each Cooper’s ligament; superior pubic ramus; obturator internus; visceral connective tissue of the vagina; urethra; bladder.

The vaginal walls are identified through blunt dissection. An assistant elevated the vaginal wall via the vaginal route. One suture was placed on each side: a single stitch through the medial portion of each Cooper’s ligament (Figure 2) and two stitches on the vaginal wall below the Foley catheter, positioned in the mid-urethral area (Figure 3). The sutures were tied with intracorporeal knots without applying excessive tension (Figure 4). The parietal peritoneum was then closed using a continuous suture. No drainage was required, and the Foley catheter was removed 24 h postoperatively.

### 2.5. Follow-Up

The follow-up period comprised evaluation at 6, 12, and 24 months after the procedure. During each visit, each patient’s data were recorded via validated questionnaires: the Urinary Distress Inventory-6 (UDI-6), the Patient Global Impression of Improvement (PGI-I), and the Incontinence Severity Index (ISI). The validated questionnaires were completed during a phone call discussion between the patient and the doctor.

The UDI-6 consists of 6 items: 1—Frequent urination, 2—Leakage related to feeling of urgency, 3—Leakage related to activity, 4—Coughing, or sneezing small amounts of leakage (drops), 5—Difficulty emptying the bladder, and 6—Pain or discomfort in the lower abdominal or genital area. Higher scores on the UDI-6 indicate higher disability. UDI-6 scores of more than 33.33 indicate higher distress caused by SUI symptoms. The total score is from 0 to 100 [13]. The PGI-I provides a measure of perceived improvement. Respondents use a 7-point Likert scale ranging from 1 (“very much better”) to 7 (“very much worse”). The PGI-I has been previously used to determine the success of incontinence procedures [14]. Consistent with the definition employed in the Value of Urodynamic Evaluation (VALUE) study, treatment success was defined as a PGI-I score of ≤2 (i.e., “very much better” or “much better”) [15].

The ISI is a simple questionnaire with only 2 questions (frequency of urine leakage and its quantity). It generates a score that categorizes patients into slight (score 1–2), moderate (score 3–6), severe (scores 8–9), or very severe (score 12) SUI [16]. The ISI was chosen because it offers a very simple and inexpensive way of calculating the severity of SUI and has been well validated by previous studies [17].

### 2.6. Data Collection and Tools

Data were collected using standardized case report forms and were subsequently organized using Microsoft Excel 2021. Patient demographics, clinical characteristics, and outcomes were systematically recorded. Subjective outcomes were assessed using validated instruments, including the UDI-6 questionnaire.

### 2.7. Statistical Analyses

Statistical analyses were performed using JASP software version 0.19.2. Descriptive statistics were employed to summarize demographic and clinical data, with results expressed as means and standard deviations for continuous variables and frequencies and percentages for categorical variables. The χ^2^ test was used for comparisons between categorical variables, while ANOVA was applied to evaluate differences between follow-up intervals for continuous data. Statistical significance was defined as *p* < 0.05.

## 3. Results

Between January 2018 and December 2022, a total of 101 patients with SUI were treated using the modified laparoscopic Burch procedure in our clinic, and a total of 83 patients completed the 2-year follow-up.

### 3.1. Age Distribution and Follow-Up Analysis

**Figure 5:**
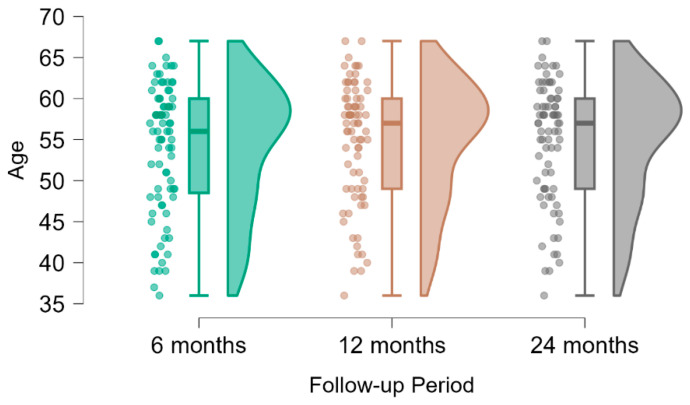
Violin and boxplot representation of age distribution across different follow-up periods, illustrating variability and central tendencies at 6, 12, and 24 months.

The mean age of participants remained stable across all follow-up intervals, highlighting the consistency of the study population. At 6 months, the mean age was 54.1 years (±7.87), increasing marginally to 54.8 years (±7.42) at both the 12- and 24-month evaluations. The minimum and maximum ages, ranging from 36 to 67 years, demonstrated a diverse cohort with a balanced representation of younger and older patients (Table 1 and Figure 5). The lack of significant changes in these demographic characteristics ensures the reliability of longitudinal comparisons within the study.

### 3.2. Subjective Efficacy of the Procedure

Patient-reported outcomes indicated a progressive improvement in perceived well-being over time. At the 6-month follow-up, 50.6% of participants reported their condition as “greatly improved”, increasing cumulatively to 66.7% by 24 months. Conversely, the proportion of patients indicating “no change” or “somewhat better” outcomes decreased over the same period, underscoring the growing efficacy of the intervention with time (Table 2). Statistical analysis using χ^2^ revealed no significant differences between intervals (*p* = 0.443), affirming the neutrality of these trends and suggesting the uniform effectiveness of the technique throughout the observation period.

The severity of urinary incontinence symptoms was markedly reduced following the intervention. The incidence of severe incontinence was notably low, with only 4.8% of patients affected at 6 months, remaining consistent at 5.1% at the 24-month mark (Table 3). This finding aligns with a high procedural success rate, as the vast majority of patients (≥94.9%) reported no severe symptoms across all follow-up intervals. These results are particularly encouraging, given the debilitating nature of severe urinary incontinence and its profound impact on quality of life.

### 3.3. Dryness Outcomes

Dryness, defined as the absence of urinary leakage, demonstrated an upward trend over time. At 6 months, 45.8% of patients reported complete dryness, with this figure rising to 55.1% at 12 months and 62.8% at 24 months (Table 4). Although these findings underscore the efficacy of the procedure in restoring continence, statistical analysis did not yield significant differences between intervals (*p* = 0.094). Nevertheless, the consistent improvement suggests a sustained therapeutic benefit, which may become statistically significant with a larger sample size or extended follow-up duration.

### 3.4. Quality-of-Life Metrics (UDI-6 Score)

The UDI-6 served as a critical metric for evaluating the subjective burden of urinary symptoms. Across all follow-up intervals, over 97% of patients achieved scores below the clinically significant threshold (<33), indicating substantial symptom relief and enhanced quality of life (Table 5). This result highlights the intervention’s effectiveness not only in addressing physical symptoms but also in mitigating their psychosocial impact.

### 3.5. Parity and Surgical Outcomes

The influence of parity on surgical outcomes was also examined. Despite the potential association between higher parity and weakened pelvic floor support, our results revealed no significant differences in outcomes across parity groups. The frequency distribution of parity was analyzed across different follow-up periods (6, 12, and 24 months) in patients who underwent the modified laparoscopic Burch procedure. At 6 months, the majority of patients had one previous childbirth (49.39%), followed by those with two (28.92%), three (13.25%), and four (3.61%) previous births. By 12 and 24 months, the distribution remained relatively stable, with 50% of patients having one childbirth, 28.21% having two, 14.10% having three, and 3.85% having four. The total number of patients decreased from 83 at 6 months to 78 at both 12 and 24 months due to loss to follow-up. Overall, the parity distribution among participants exhibited consistency throughout the study period. Patients with multiple childbirths demonstrated similar improvements to those with fewer or no prior deliveries, suggesting that the operative technique is robust and applicable across a diverse range of pelvic floor conditions. This finding broadens the potential applicability of the intervention and supports its use in clinical settings where parity is a consideration.

### 3.6. Influence of Age on Outcomes

**Figure 6:**
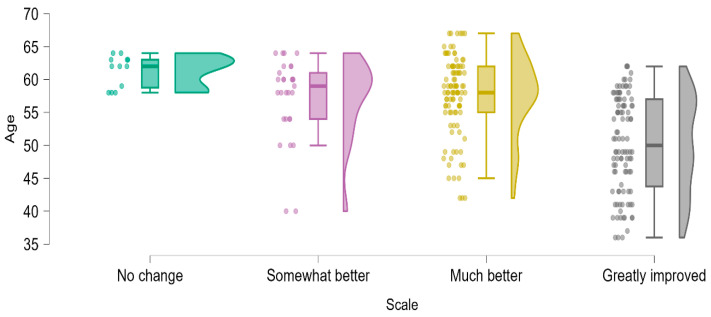
Violin and boxplot representation of age distribution across different levels of symptom improvement, providing a visual comparison of age-related differences in treatment response.

Age emerged as a significant factor influencing patient-reported outcomes. Stratification by subjective improvement categories revealed a clear trend: younger patients consistently reported more favorable outcomes compared to their older counterparts. The mean age of patients reporting “no change” was 61.3 years, decreasing progressively to 57.1 years for “somewhat better”, 57.5 years for “much better”, and 50.2 years for “greatly improved” (Table 6, Figure 6). This pattern may be attributed to biological factors such as greater tissue elasticity, improved healing capacity, and fewer comorbidities in younger patients. Conversely, older individuals may experience diminished outcomes due to age-related factors such as reduced collagen synthesis, altered pelvic floor dynamics, and the presence of chronic conditions.

### 3.7. Postoperative Voiding Difficulties

The analysis of voiding difficulties revealed an extremely low incidence of the condition across the 24-month follow-up period, with only one patient experiencing this complication at 6 months and no cases reported thereafter (Table 7 and Table 8). A Chi-squared test for the distribution of voiding difficulties over the three follow-up intervals indicated no statistically significant differences, with a *p*-value of 0.389 and a Chi-squared value of 1.887 for two degrees of freedom. These results suggest that the operative technique employed demonstrates excellent safety in preventing postoperative voiding difficulties and maintaining a stable and favorable profile throughout the observation period. This finding aligns with the broader data on procedure-related complications, further supporting the intervention’s clinical utility.

**Figure 7:**
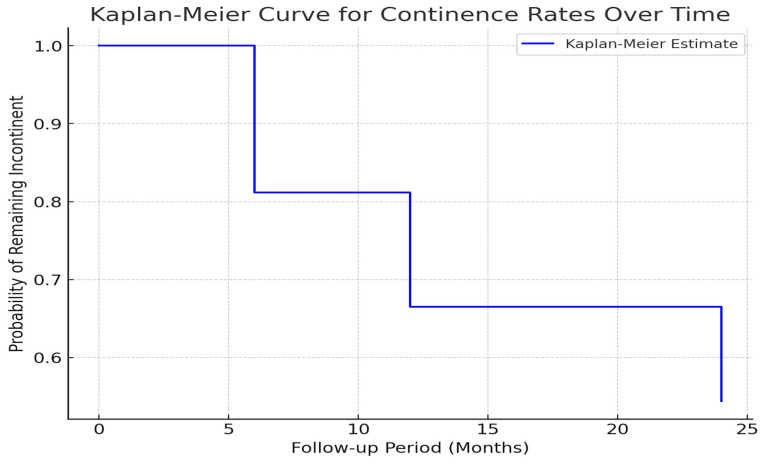
Kaplan–Meier survival curve illustrating the probability of remaining incontinent over the follow-up period (6, 12, and 24 months). The stepwise decline represents the proportion of patients achieving continence at each interval.

The continence rates following the modified laparoscopic Burch procedure were evaluated using Kaplan–Meier survival analysis, allowing for a longitudinal assessment of the probability of remaining incontinent across different follow-up intervals. Figure 7 presents the Kaplan–Meier curve, illustrating the cumulative proportion of patients achieving continence over time. The stepwise nature of the graph reflects the sequential improvement in continence rates, with noticeable declines at 6, 12, and 24 months postoperatively, corresponding to patients achieving dryness.

At the 6-month follow-up, a substantial proportion of patients remained incontinent, reflecting the early postoperative adaptation phase. By 12 months, a marked reduction in incontinence was observed, aligning with patient-reported outcomes indicating progressive symptom relief. At 24 months, continence rates continued to improve, underscoring the long-term efficacy of the modified technique. The Kaplan–Meier estimate provides a robust statistical framework for evaluating the durability of continence outcomes while accounting for censored observations, such as patients lost to follow-up.

These findings reinforce the clinical utility of the modified laparoscopic Burch colposuspension as a viable alternative for patients requiring surgical intervention for stress urinary incontinence. The progressive improvement in continence over time suggests a sustained therapeutic benefit, which may be further validated through extended follow-up periods and larger sample sizes in future studies.

## 4. Discussion

Within the United States, the FDA has proposed elevating the risk classification of urogynecological meshes. Amid growing criticism of transvaginal synthetic meshes, patients are increasingly seeking alternative treatments for SUI. Consequently, the Burch colposuspension is experiencing a resurgence in popularity [18,19,20].

Originally designed as an open surgical procedure, the Burch colposuspension has been shown in several studies to be effectively performed laparoscopically, reducing procedure-related trauma. The traditional approach involves placing two to four sutures lateral to the urethra and bladder neck on each side. This technique not only limits urethral hypermobility but also alters the angle between the urethra and bladder neck [18].

Considering the standard female urethral length of 3–4 cm, placing two sutures as in the standard Burch technique may increase the risk of altering the anatomy of the bladder neck angle, potentially leading to voiding difficulties [18]. In the modified Burch technique we performed, a single suture is placed on each side caudal to the Foley catheter. This approach stabilizes the middle third of the urethra, limiting hypermobility without altering the angle between the urethra and bladder neck. It creates stability in the same region as a suburethral sling, achieving a similar effect without the use of polypropylene material [7].

Mastering the robotic-assisted approach to the Burch procedure offers numerous advantages, including enhanced visualization and precise dissection of the bladder edge, retropubic space, and surrounding vessels, and creating a better setting for the stitches. Additionally, the robotic instruments provide a greater range of motion compared to laparoscopic tools. This approach improves both the ease and safety of the procedure, solidifying its place in the toolbox of the modern gynecologist [21].

Persson et al. made a randomized comparison of one or two sutures on each side of the urethra in laparosocopic Burch colposuspension. The analysis showed a lower subjective cure rate with one suture on each side of the urethra (65%) compared with two sutures on each side of the urethra (89%) [22]. Conversely, Polascik et al. found a higher cure rate of 83% using one stitch on each side of the urethra laparoscopic Burch colposuspension [23]. Subsequently, in 1998, Miannay et al. performed a retrospective study comparing the data of 144 patients who underwent laparoscopic Burch or open Burch procedures, finding no statistical difference between the cure rate of the two procedures (68% vs. 64%, *p*-value non-significant) after a 24-month follow-up. The authors performed laparoscopic surgeries with only one stitch on each side of the urethra, whereas the open technique used two classic stitches on each side [24,25].

Regarding cure rate after the TOT procedure, Bandarian et al. reported a higher complete cure rate in the TOT group, with 90.3% [26], and in a prospective trial by Sivaslioglu et al. [27], the cure rates of SUI after TOT at one year were 85.7% and 87.5% at the two-year follow-up (32 patients of 49 were available). Asicioglu et al. reported an objective cure rate of 77.5% and a subjective cure rate of 81.7% in their TOT group (272 patients), with a longer, 5-year period of follow-up [28]. Regarding the cure rate after traditional Burch colposuspension, the cure rate after 24 months of follow-up was 86% in the Liapis et al. study [29], and El-Barky et al. reported in their study a 72% cure rate [30].

Success defined by the PGI-I as “very much improved” and “much improved” in the Burch colposuspension group was reported by 84.1% (243 of 289 patients) in the Karmakar et al. study [31], and 72% of subjects in the laparoscopic Burch group reported they were “much better” or “very much better” compared with before their surgery, while 7% and 4% reported they were “much worse” or “very much worse”, respectively, on the PGI-I questionnaire in the study by Jelovsek et al. [32].

Jelovsek et al. also reported in their study, based on the ISI questionnaire, that 22% (6/28) of subjects in the laparoscopic Burch group had moderate to severe incontinence of urine 4–8 years after surgery. Dryness was reported in their study by 12 of 28 (43%) patients [32]. In our study, at the 24-month follow-up, 62.8% patients reported complete dryness after the modified laparoscopic Burch procedure.

In our study, at all follow-up intervals, more than 97% of patients achieved scores below the clinically significant threshold (<33) using UDI-6, demonstrating significant symptom relief and improved quality of life. Also, Jelovsek et al. reported a significantly improved quality of life on UDI-6 in the laparoscopic Burch group at 1–2 years follow-up. This result highlights the effectiveness of the intervention not only in addressing physical symptoms but also in mitigating their psychosocial impact [32].

Regarding the cure rate after the modified laparoscopic Burch procedure at the 6-month follow-up, 50.6% of participants reported their condition as “greatly improved”, increasing cumulatively to 66.7% by 24 months. In their research, Conrad et al. reported higher subjective success rates, with 78.1% of patients reporting no symptoms of SUI at a mean follow-up of 50.6 months and 12.4% reporting significantly improved symptoms in the laparoscopic Burch group [33]. Yang et al. described their high results in the laparoscopic Burch group, with 116 of 155 women available at 1-year follow-up, yielding an objective cure rate of 94.8% (110/116) and a subjective cure rate of 95.7% (110/116) [34]. Hong et al. reported a 72.1% cure rate in their laparoscopic Burch group [35]. The high cure rate can be explained by the short follow-up period.

Compared to the traditional Burch procedure, with a high incidence of voiding difficulties, reported in up to 20% of cases, as well as the occurrence of de novo urgency, the modified technique aligns with the principles of anatomical restoration outlined in Petros’ Integral Theory [9,11,19]. This alignment is reflected in the lower rates of de novo urgency and voiding difficulties observed in patients treated with the modified technique.

Following anti-incontinence surgery, voiding dysfunction can occur as a known complication, often resulting from overcorrection of the urethra due to excessive elevation of the bladder neck. Previous studies have reported that postoperative voiding difficulties and urinary retention affect up to 20% of patients after Burch colposuspension, 10% after pubovaginal sling, 20% after transvaginal tape (TVT), and 20% after transobturator tape (TOT) [36]. In a comparison between TVT and TOT, the obturator route was found to be associated with a lower incidence of bladder injury and voiding difficulty [37,38,39].

Conrad et al. reported that the most common significant perioperative complication was postoperative voiding dysfunction, although this occurred in only 10% of patients [33]. Lose et al. found that Burch colposuspension could alter the original micturition pattern, potentially introducing an element of obstruction that disrupts the balance between voiding forces and outflow resistance. This disruption may lead to both immediate postoperative and long-term voiding difficulties [40]. Karmakar et al. reported that the incidence of long-term severe voiding difficulty needing self-catheterization was 0.3% in Burch colposuspension [31]. Lemack et al. reported their results from a prospective randomized trial after Burch colposuspension. They concluded that normal preoperative urodynamic testing does not predict postoperative voiding dysfunction among women undergoing surgery for SUI [41].

Several studies have suggested that poor detrusor contraction or voiding by Valsalva maneuver, characterized by intra-abdominal pressures exceeding 10 cm H_2_O during voiding and detrusor pressures less than 15 cm H_2_O, are associated with impaired postoperative voiding in women who undergo incontinence surgery. Bhatia and Bergman found that women who had adequate detrusor contraction and normal flow rates on pressure-flow voiding studies preoperatively were able to resume spontaneous voiding by the seventh postoperative day after Burch colposuspension [42,43,44].

On the other hand, Weinberger et al. demonstrated no significant correlation between preoperative uroflowmetry findings and the development of postoperative voiding dysfunction [45].

Several studies have identified risk factors for postoperative voiding dysfunction, including demographic factors, voiding parameters, and anatomical factors.

Strength of our study: Satisfaction assessment using the PGI-I, which is a crucial outcome measure.

Limitations of the study: The study included a relatively small cohort of patients, which may limit the generalizability of the findings to a broader population.

Another limitation of the study is the relatively short follow-up period. Although a 24-month follow-up provides valuable insights, longer-term outcomes beyond 2 years remain unknown, particularly concerning the durability of symptom relief and potential late-onset complications. Although postoperative voiding difficulties were minimal in our cohort, long-term risks such as de novo urgency, urge incontinence, or POP should be considered. Reports from similar surgical techniques indicate that a small subset of patients may develop these complications years after surgery, possibly due to changes in pelvic floor dynamics, hormonal shifts, or progressive tissue laxity.

Future research: Larger, multicenter studies with diverse populations would provide a broader understanding of the procedure’s efficacy and safety.

## 5. Conclusions

The modified laparoscopic Burch colposuspension demonstrated consistent efficacy, with significant improvements in urinary continence, symptom severity, and quality of life over the 24-month follow-up period. The proportion of patients achieving “complete dryness” increased steadily, highlighting the procedure’s long-term benefits. Although trends in improvement were evident, some results, such as differences in dryness rates or subjective improvements, did not achieve statistical significance. This highlights the need for larger sample sizes or extended follow-up in future studies. This study reinforces the role of modified Burch colposuspension as an effective and safe intervention for managing urinary incontinence, offering durable benefits and improved quality of life for patients.

## Figures and Tables

**Table 1:** Descriptive statistics summarizing the age distribution of participants at different follow-up intervals (6, 12, and 24 months), including mean age, standard deviation, and range.

Descriptive Statistics			
	Age		
	6 Months	12 Months	24 Months
Valid	83	78	78
Mean	54.108	54.846	54.846
Std. Deviation	7.871	7.419	7.419
Minimum	36.000	36.000	36.000
Maximum	67.000	67.000	67.000

**Table 2:** Distribution of symptom improvement across follow-up periods, based on patient-reported outcomes, categorized into four levels: no change, somewhat better, much better, and greatly improved.

Follow-Up Period	Scale	Frequency	Percent	Cumulative Percent
6 months	No change	4	4.819	4.819
	Somewhat better	7	8.434	13.253
	Much better	30	36.145	49.398
	Greatly improved	42	50.602	100.000
	Total	83	100.000	
12 months	No change	3	3.846	3.846
	Somewhat better	11	14.103	17.949
	Much better	32	41.026	58.974
	Greatly improved	32	41.026	100.000
	Total	78	100.000	
24 months	No change	5	6.410	6.410
	Somewhat better	11	14.103	20.513
	Much better	36	46.154	66.667
	Greatly improved	26	33.333	100.000
	Total	78	100.000	

**Table 3:** Proportion of patients experiencing severe incontinence at 6, 12, and 24 months postoperatively, with cumulative percentages showing overall trends.

Follow-Up Period	Severe Incontinence	Frequency	Percent	Cumulative Percent
6 months	No	79	95.181	95.181
	Yes	4	4.819	100.000
	Total	83	100.000	
12 months	No	74	94.872	94.872
	Yes	4	5.128	100.000
	Total	78	100.000	
24 months	No	74	94.872	94.872
	Yes	4	5.128	100.000
	Total	78	100.000	

**Table 4:** Proportion of patients achieving dryness at each follow-up interval, illustrating the effectiveness of the surgical intervention over time.

Follow-Up Period	Dryness	Frequency	Percent	Cumulative Percent
6 months	No	38	45.783	45.783
	Yes	45	54.217	100.000
	Total	83	100.000	
12 months	No	43	55.128	55.128
	Yes	35	44.872	100.000
	Total	78	100.000	
24 months	No	49	62.821	62.821
	Yes	29	37.179	100.000
	Total	78	100.000	

**Table 5:** Urinary Distress Inventory (UDI-6) scores stratified by follow-up period, indicating the proportion of patients with significant urinary distress (>33) versus those with lower distress scores.

Follow-Up Period	UDI 6	Frequency	Percent	Cumulative Percent
6 months	>33	2	2.410	2.410
	<33	81	97.590	100.000
	Total	83	100.000	
12 months	>33	2	2.564	2.564
	<33	76	97.436	100.000
	Total	78	100.000	
24 months	>33	2	2.564	2.564
	<33	76	97.436	100.000
	Total	78	100.000	

**Table 6:** Comparison of age distribution among patients reporting different levels of symptom improvement, highlighting mean age variations across response categories.

*Descriptive Statistics*				
	Age			
	No Change	Somewhat Better	Much Better	Greatly Improved
Valid	12	29	98	100
Mean	61.333	57.172	57.520	50.160
Std. Deviation	2.387	6.297	6.069	7.315
Minimum	58.000	40.000	42.000	36.000
Maximum	64.000	64.000	67.000	62.000

**Table 7:** Data represent the observed frequency of voiding difficulties across follow-up periods. The Chi-squared test indicated no statistically significant differences between intervals (*p* = 0.389).

Contingency Tables			
	Voiding Difficulties		
Follow-Up Period	No	Yes	Total
6 months	82	1	83
12 months	78	0	78
24 months	78	0	78
Total	238	1	239

**Table 8:** Frequencies and percentages represent the distribution of voiding difficulties across follow-up periods. Valid Percent excludes missing data. No cases of voiding difficulties were reported after the 6-month follow-up.

Follow-Up Period	Voiding Difficulties	Frequency	Percent	Cumulative Percent
6 months	No	82	98.795	98.795
	Yes	1	1.205	100.000
	Total	83	100.000	
12 months	No	78	100.000	100.000
	Yes	0	0.000	100.000
	Total	78	100.000	
24 months	No	78	100.000	100.000
	Yes	0	0.000	100.000
	Total	78	100.000	

## Data Availability

Data are contained within the article.
